# The AC2 Protein of a Bipartite Geminivirus Stimulates the Transcription of the BV1 Gene through Abscisic Acid Responsive Promoter Elements

**DOI:** 10.3390/v12121403

**Published:** 2020-12-07

**Authors:** Rong Sun, Junping Han, Limin Zheng, Feng Qu

**Affiliations:** Department of Plant Pathology, Ohio Agricultural Research and Development Center, The Ohio State University, Wooster, OH 44691, USA; sun.1207@buckeyemail.osu.edu (R.S.); han.393@osu.edu (J.H.); zheng.1811@osu.edu (L.Z.)

**Keywords:** geminivirus, bipartite begomovirus, transcriptional trans-activation, abscisic acid, promoter motifs, AC2, BV1, mungbean yellow mosaic virus

## Abstract

Geminiviruses possess single-stranded, circular DNA genomes and control the transcription of their late genes, including BV1 of many bipartite begomoviruses, through transcriptional activation by the early expressing AC2 protein. DNA binding by AC2 is not sequence-specific; hence, the specificity of AC2 activation is thought to be conferred by plant transcription factors (TFs) recruited by AC2 in infected cells. However, the exact TFs AC2 recruits are not known for most viruses. Here, we report a systematic examination of the BV1 promoter (P_BV1_) of the mungbean yellow mosaic virus (MYMV) for conserved promoter motifs. We found that MYMV P_BV1_ contains three abscisic acid (ABA)-responsive elements (ABREs) within its first 70 nucleotides. Deleting these ABREs, or mutating them all via site-directed mutagenesis, abolished the capacity of P_BV1_ to respond to AC2-mediated transcriptional activation. Furthermore, ABRE and other related ABA-responsive elements were prevalent in more than a dozen Old World begomoviruses we inspected. Together, these findings suggest that ABA-responsive TFs may be recruited by AC2 to BV1 promoters of these viruses to confer specificity to AC2 activation. These observations are expected to guide the search for the actual TF(s), furthering our understanding of the mechanisms of AC2 action.

## 1. Introduction

Plant viruses of the family *Geminiviridae* are among the most important pathogens of crop plants worldwide [1,2,3,4]. Examples of agriculturally devastating geminiviruses include the tomato yellow leaf curl virus (TYLCV) and its close relatives, the maize streak virus (MSV) and numerous variants of the African cassava mosaic virus [5,6]. An increasing number of geminiviruses have been discovered during the last decade, with their geographic distribution steadily broadening, thanks to the global climate change that permits their insect vectors to flourish in previously hostile territories [7]. As a result, more research is urgently needed in order to inform a more effective management of these viruses.

Geminiviruses all have characteristic, two-headed particles that encapsidate single-stranded (ss), covalently circularized DNA genomes, mostly under three kilobases (kb) in size [2]. These small ssDNA genomes nevertheless harbor multiple open reading frames (ORFs) on both the genome (or viral, V) strand and the strand complementary to the genome (C strand). Many of these ORFs overlap with each other, allowing for up to six different viral proteins per genome segment. While most geminiviruses have just one circular ssDNA genome segment, some viruses belonging to the genus Begomovirus, known as bipartite begomoviruses, have a second genome segment that encode additional important proteins [7,8]. Bipartite begomoviruses share similar coding strategies. Typically, one of the genome segments, designated DNA-A, encodes five proteins, with one of them, AV1, on the V strand and the remaining four, AC1-4, on the C strand. The other genome segment, DNA-B, encodes just two proteins: BV1 on the V strand and BC1 on the C strand. AC1, also known as Rep, is the earliest expressing viral protein and the only viral protein absolutely needed for the replication of the viral genome [1,2,9,10]. It functions by coercing the host cell DNA replication machinery into replicating the viral genomes in the host cell nucleus. AC2, AC3, and AC4 are also early expressing, with AC2 serving as the transcriptional trans-activator (TrAP) of late genes. AC3 is a replicational enhancer (REn), and AC4 acts as a multifunctional symptom determinant [2,3,11,12]. AV1, BC1, and BV1 function as the capsid protein (CP), movement protein (MP), and nuclear shuttle protein (NSP), respectively.

The mechanism of the AC2-mediated trans-activation of late genes, especially those encoded by DNA-B, is of great research and application interests, because, once understood, it offers the potential of engineering begomovirus resistance that is activated by the invading virus itself. For example, antiviral defense factors, such as resistance genes (R genes) or silence-inducing double-stranded (ds) RNA, can be placed under the control of promoters activatable by AC2, so that they would only express when the corresponding virus replicates in the cell to produce AC2. The particular mechanism adopted by the tomato golden mosaic virus (TGMV) has been extensively examined by Sunter and colleagues. Sunter and Bisaro were among the first to show that AL2 of TGMV, which corresponds to AC2 in other bipartite begomoviruses, activated BR1 (BV1 in other viruses) expression at the transcription level [13,14]. Note that, although the AC2/AL2 proteins of different bipartite begomoviruses share important structural features characteristic of transcription factors (TFs), their interaction with DNA, including ssDNA, did not display any sequence specificity [15,16,17]. It has hence been hypothesized that AC2 exerts its trans-activation role by recruiting various TFs of the host plant, with the latter conferring the needed DNA-binding specificity. Consistent with this hypothesis, the AL2 of TGMV was found to interact with the PEAPOD2 (PPD2) protein of Arabidopsis [18]. PPD2 was also shown to bind to the promoter of BR1, hence bridging AL2 and the BR1 promoter, facilitating the transcriptional activation of BR1 [19].

The mungbean yellow mosaic virus (MYMV) is a bipartite begomovirus that infects important legume crops such as mungbean, soybeans, and lima beans [20]. Its two genome segments are approximately 2730 and 2660 nucleotides (nt) in size, respectively, encoding a similar set of viral proteins as TGMV, albeit with notable differences. Compared to TGMV, MYMV and other so-called “Old World” begomoviruses possess two additional ORFs, AV2 and BV2, although the functions of the putative AV2 and BV2 proteins remain enigmatic [21,22]. 

The current study set out to determine how expression of the BV1 gene of MYMV reacts to AC2-mediated transcriptional activation. Our study was inspired by a previous report [21], which mapped the AC2-responsive region of the BV1 promoter (designated P_BV1_ hereafter) to nt positions 26–419 (394 nt in total) of MYMV DNA-B. Based on the results obtained by Sunter and colleagues using the TGMV system, we hypothesized that the 394-nt P_BV1_ contained conserved promoter motifs recognizable by certain plant TFs that also interacted with AC2. Here, we report a systematic dissection of MYMV P_BV1_, leading to the identification of multiple sequence motifs previously shown to be critical for plant responses to abscisic acid (ABA). 

ABA is a well-characterized plant hormone that plays critical roles in multiple stages of the plant life cycle, including maintaining seed dormancy and spearheading plant responses to drought and cold stresses during vegetative growth [23,24]. ABA exerts these roles through multiple signal transduction pathways that enlist numerous ABA-responsive TFs. These TFs act on ABA-responsive promoters containing conserved sequence motifs known as the ABA-responsive element (ABRE) and coupling element 1 and 3 (CE1 and CE3), among others [25,26,27]. Here, we report that MYMV P_BV1_ contains three abscisic acid (ABA)-responsive elements (ABREs) within the first 70-nt region. Eliminating these ABRE motifs through either deletion or site-directed mutagenesis caused dramatic losses in the P_BV1_ responsiveness to AC2-mediated activation. ABRE, as well as CE1 and CE3, are also present in more than a dozen bipartite begomoviruses of the Old World lineage. These results strongly suggest that one or more ABA-responsive TFs are probably recruited by AC2 to confer the DNA-binding specificity needed for the transcriptional activation of MYMV P_BV1_. 

## 2. Materials and Methods 

### 2.1. Constructs

The Core35S-GFP construct was based on pAI101, a binary vector modified from pCambia1300 [28,29]. An expression cassette consisting of the core 35S promoter (the last 99 nt of the cauliflower mosaic virus (CaMV) 35S promoter), the EGFP cDNA, and the poly-A signal of CaMV 35S RNA was inserted into pAI101 at the PstI site to create Core35S-GFP. The Core35S-GFP construct was then used as the backbone to create constructs in which the Core35S promoter was replaced by the sequences of the MYMV BV1 promoter, its counterpart in the soybean yellow mosaic virus (SYMV), and their mutated derivatives. The corresponding DNA fragments were custom-synthesized by Integrated DNA Technologies (IDT, Coralville, IA, USA) as gBlocks fragments and used to replace the Core35S sequence via Gibson Assembly cloning (New England Biolabs, Ipswich, MA, USA). The AC2-expressing construct was also made in pAI101, in which the AC2 cDNA was preceded by the CaMV 35S promoter with double enhancers (2X35S), plus a translational enhancer derived from the tobacco etch virus (TEV TE) [28,29,30]. The identity of all constructs was verified with Sanger sequencing. 

### 2.2. Agro-Infiltration

Upon verification, the constructs were introduced into the *Agrobacterium tumefaciens* strain C58C1 with electroporation [30]. In most experiments, various combinations of *Agrobacterium* suspensions were mixed together and delivered into *Nicotiana benthamiana* leaves as described [28,29,30,31,32]. A p19-expressing *Agrobacterium* strain was included in all combinations to alleviate RNA silencing-mediated mRNA degradation. In experiments testing the promoter responsiveness to abscisic acid (ABA) treatment, a 50-mM ABA stock solution was prepared by dissolving ABA powder (GoldBio, St. Louis, MO, USA) in dimethyl sulfoxide (DMSO). It was then added to the *Agrobacterium* suspensions to a final concentration of 20 µM. Accordingly, the same *Agrobacterium* suspensions containing 1:2500 diluted DMSO were used as the controls for the ABA treatment. 

### 2.3. Protein Extraction and Western Blotting

Proteins were extracted from the agro-infiltrated *N. benthamiana* leaves following a published protocol [28] and subjected to Western blot detection of the GFP protein with a GFP antibody. The rabbit polyclonal GFP antibody was purchased from Invitrogen (Carlsbad, CA, USA). The HRP-conjugated anti-rabbit secondary antibody was purchased from Abcam (Cambridge, MA, USA). 

### 2.4. Confocal Microscopy

Confocal microscopic observations were carried out using a Leica Confocal microscope (TCS SP5) at the Molecular and Cellular Imaging Center, the Ohio Agricultural Research and Development Center, The Ohio State University. The samples were mounted under a 10× lens, and the GFP fluorescence was excited with a 488-nm Argon blue laser. The images were captured with an emission wavelength of 517 nm. The resolution pixels of the original images were 1024 × 1024. 

## 3. Results

### 3.1. The First 100 nt of MYMV P_BV1_ Contains AC2-Responsive DNA Element(s)

**Figure 1:**
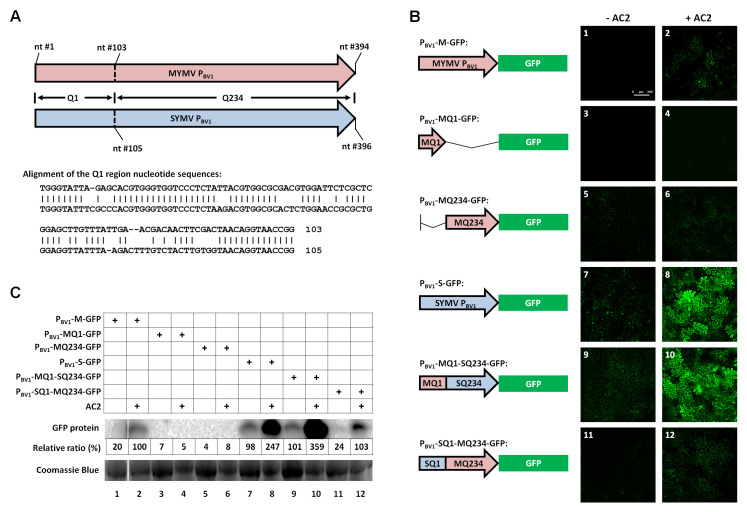
The first quarter of the mungbean yellow mosaic virus (MYMV) BV1 promoter determines the specificity of AC2-mediated transcriptional activation. (**A**) Schematic representation of the MYMV and soybean yellow mosaic virus (SYMV) P_BV1_ promoters and the nucleotide (nt) sequence alignment, showing a high similarity between the two promoters within the first 100 nt of the approximately 400-nt promoters. Q1: The first quarter, which is 103 and 105 nt in the MYMV and SYMV, respectively. Q234: The remaining three quarters of the promoters that are highly dissimilar. (**B**) Confocal microscope images (Bar = 200 µM) of *Nicotiana benthamiana* cells showing the relative GFP expression levels driven by the MYMV P_BV1_, its deletion mutants (MQ1 and MQ234), SYMV P_BV1_, and two chimeric promoters (MQ1-SQ234 and SQ1-MQ234). Illustrations of the constructs are shown to the left, and the corresponding GFP expression levels in the absence (odd-numbered panels) and presence (even-numbered panels) of AC2 are on the right. Note that the microscope setting was the same for all images. (**C**) Western blot detection of the GFP protein in the agro-infiltrated leaves. The lane numbers correspond to the panel numbers in (**B**). To minimize the variations, each leaf sample consisted of four separate leaf sections taken from four different plants. The relative ratios were the averages of two independent sets of experiments, with the band intensity value of P_BV1_ + AC2 set at 100%.

We described in the Introduction that AC2 of MYMV, similar to AL2 of TGMV, probably activated the transcription of P_BV1_ indirectly by recruiting one or more plant TFs to confer the DNA-binding specificity. Since different families of TFs are known to bind to different conserved promoter motifs, TFs that bridge AC2 and P_BV1_ could be inferred from the enrichment of a specific class of conserved DNA sequence motifs in MYMV P_BV1_. To begin to delineate the conserved DNA motifs in MYMV P_BV1_, we took advantage of the study of Shivaprasad and colleagues [21], which mapped MYMV P_BV1_ to a 394-nt region (nt position 26-419 of MYMV DNA-B; Figure 1A). Since the MYMV is very closely related to the soybean yellow mosaic virus (SYMV), another Old World bipartite begomovirus [33], we first compared the MYMV P_BV1_ with its counterpart in the SYMV. Surprisingly, significant sequence similarities between these two BV1 promoters were found only within the first 103-nt (105 nt in the SYMV) region, which we will call Q1 (meaning the first one-quarter of the P_BV1_) from here on (Figure 1A). By contrast, the remaining three-quarters (Q234) of the promoters diverged dramatically from each other.

The limited range of the promoter sequence similarity, coupled with the near identity of the AC2 proteins of the two viruses (132 of the entire 135 amino acid residues are identical), led us to hypothesize that the Q1 region of the MYMV P_BV1_ contained the specific sequence motif(s) needed for TF binding and, hence, AC2-mediated transcriptional activation. This hypothesis was tested by fusing various portions and combinations of MYMV and SYMV P_BV1_ with a GFP reporter (Figure 1B). The resulting constructs were delivered into the cells of *N. benthamiana* plants via agro-infiltration [30]. The expression of GFP from these constructs, with or without MYMV AC2, was assessed with confocal microscopy, as well as Western blot detection of the GFP protein. Note that MYMV AC2 has an additional function: suppression of RNA silencing [22]. The silencing suppression activity of AC2 can lead to mRNA stabilization, which can be mistaken as increased mRNA transcription due to AC2-mediated transcriptional activation. To neutralize this effect, we included another construct that expresses p19, a much stronger silencing suppressor [34], in all treatments. 

As seen in Figure 1B, panel 1, GFP fluorescent cells were undetectable under a confocal microscope when the MYMV P_BV1_ (P_BV1_-M) promoter was used to drive the GFP transcription without AC2. By contrast, green fluorescent cells were numerous and easily detectable when AC2 was included (Figure 1B, panel 2). These results indicated that AC2 activated mRNA transcription from P_BV1_-M, and the *N. benthamiana*-based system recapitulated the transcriptional activation activity of AC2. When assessed with Western blotting, AC2-mediated transcriptional activation caused the GFP protein level to increase by approximately five-fold (Figure 1C, lanes 1 and 2). Moreover, both the Q1 and Q234 portions of P_BV1_ were needed for this effect, because neither alone responded to AC2 activation (Figure 1B, panels 3–6 and Figure 1C, lanes 3–6). The need for Q234 was not surprising, because it contained the essential TATA box [21]. Interestingly, the P_BV1_ of the SYMV (P_BV1_-S) drove a substantially higher level of basal transcription than the P_BV1_-M (approximately five-fold; Figure 1B, panels 1 and 7 and Figure 1C, lanes 1 and 7) but, notably, a smaller magnitude (2.5 times) of transcriptional stimulation by AC2 of the MYMV (Figure 1B, panels 7 and 8 and Figure 1C, lanes 7 and 8). Exchanging the Q1 and Q234 portions of the two P_BV1_ promoters strongly suggested that the increased basal transcription was attributable to the Q234 of the SYMV, whereas the AC2-responsive element(s) likely resided in the highly homologous Q1 portion of the two promoters (Figure 1B, panels 9–12 and Figure 1C, lanes 9–12). 

### 3.2. The First 73 nt of the MYMV P_BV1_ is Sufficient to Accommodate Specific AC2 Activation 

**Figure 2:**
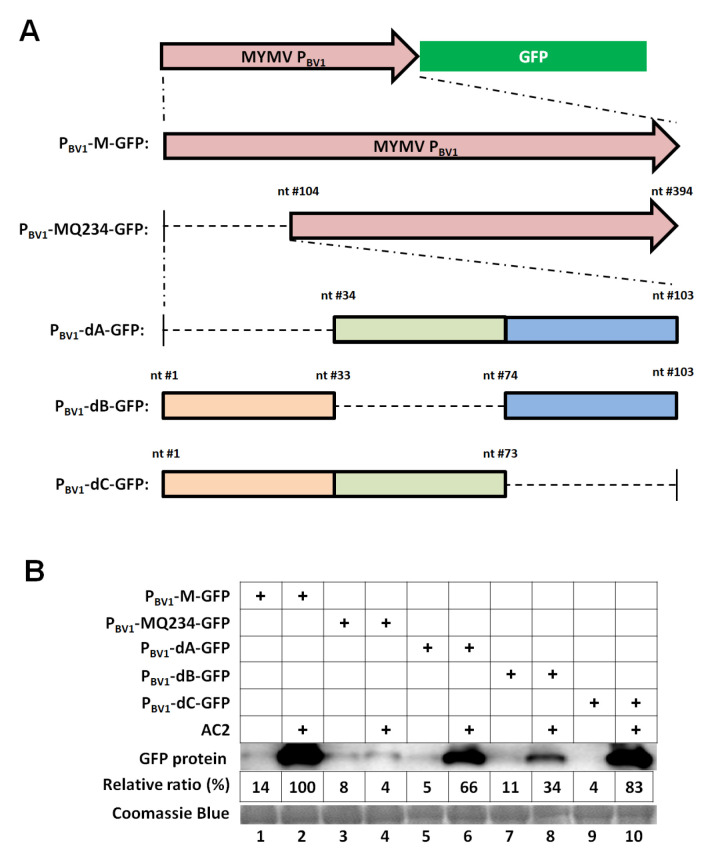
The first 73 nt of the MYMV P_BV1_ contains key promoter element(s) needed for AC2-mediated transcriptional activation. (**A**) Diagrams of three deletion mutants of the MYMV P_BV1_. All deletions were 40 nt or smaller, with their respective boundaries shown. (**B**) Western blot detection of the GFP protein in the agro-infiltrated leaves harboring the various deletion mutants. Each protein sample was extracted from four separate leaf sections infiltrated with the same *Agrobacterium* suspension, taken from four different plants. The relative ratios were averages of two independent sets of experiments, with the band intensity value of P_BV1_ + AC2 set at 100%.

In order to identify the specific DNA sequence motifs within the Q1 portion of the MYMV P_BV1_, we generated three consecutive deletions within this region (Figure 2A), with nt #1–33 (33 nt), 34–73 (40 nt), and 74–103 (30 nt) deleted in mutants dA, dB, and dC, respectively. As shown in Figure 2B, mutants dA and dB caused the AC2-dependent transcription to decrease by approximately 30% and 60%, respectively, whereas mutant dC retained more than 80% of the AC2-dependent transcription. These results pointed to the first 73-nt region of the MYMV PBV1 as the highly critical region needed for AC2-mediated transcriptional activation.

### 3.3. Three ABREs within the First 73 nt of the MYMV P_BV1_ Cooperatively Mediate Transcriptional Activation by AC2

**Figure 3:**
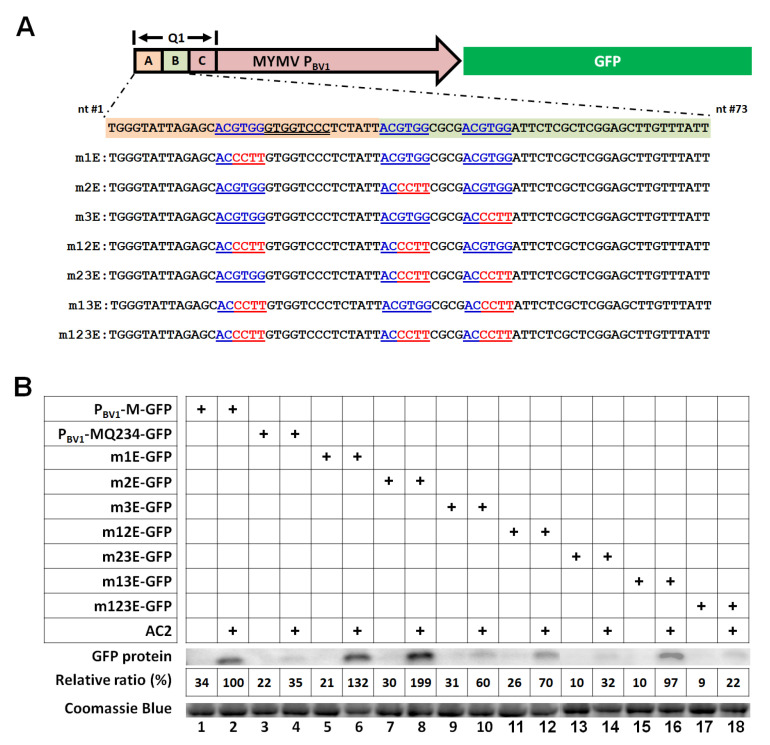
The abscisic acid (ABA)-responsive elements (ABREs) in the MYMV P_BV1_ are needed for AC2-mediated activation. (**A**) Top: Schematic representation of the P_BV1_-GFP construct. The boundaries of the Q1 section are denoted with two vertical bars, and the three regions (A, B, and C) subjected to deletions in Figure 2 are highlighted with shades of different colors. Bottom: The sequences of the A and B regions, along with that of seven mutants in the three ABREs, are variously mutated, are shown. The three ABRE motifs are underlined and highlighted in blue fonts. The conserved late element (CLE) motif is highlighted with a double underline. The mutated nt (GTGG → CCTT) are in red fonts. (**B**) Western blot detection of the GFP protein in the agro-infiltrated leaves harboring the various mutants. Each protein sample was extracted from four separate leaf sections infiltrated with the same *Agrobacterium* suspension, taken from four different plants. The relative ratios were averages of two independent sets of experiments, with the band intensity value of P_BV1_ + AC2 set at 100%.

We next asked whether this 73-nt region of the MYMV P_BV1_ contained any known sequence motifs of common TFs. Intriguingly, this relatively short region contained three ABREs, with the ACGTGG consensus [26,35], that were easily identifiable with a mere visual inspection (Figure 3A). Furthermore, the fact that the first ABRE lied within the dA region, whereas the remaining two within the dB region, was consistent with Figure 2 showing a larger impact of the dB deletion than the dA deletion. To evaluate the contribution of each of the three ABREs to AC2-mediated transcriptional activation of the P_BV1_ promoter, we then systemically mutated these ABREs, alone and in combination, by changing the “GTGG” residues to “CCTT” (Figure 3A). As shown in Figure 3B, while mutating the first or second ABRE alone appeared to enhance the AC2-mediated activation (lanes 5-8), mutating both of them reduced the AC2 effect by 30% (lanes 11 and 12). Mutations within the third ABRE reduced the AC2 effect by 40% (lanes 9 and 10), and combining them with mutations in the second ABRE further diminished the AC2 effect by 30% (lanes 13 and 14). While mutating both the first and third ABREs did not seem to hinder the AC2 action, it did negate the stimulative effect of mutations in the first ABRE (lanes 15 and 16). Finally, simultaneously mutating all three ABREs abolished the AC2 effect to the same extent as Q234, in which the entire Q1 region was deleted. Together, these results indicated that the three ABREs functioned cooperatively to accommodate the AC2-mediated transcriptional activation of the P_BV1_ promoter. 

### 3.4. BV1 Promoters of Many Old World Bipartite Begomoviruses Contain ABREs and Other ABA-Responsive Promoter Motifs

Having established that the three ABREs in the MYMV P_BV1_ functioned cooperatively to confer specificity to the AC2-mediated transcriptional activation, we wondered whether the BV1 promoters of other bipartite begomoviruses also contained ABRE or other promoter motifs involved in plant responses to ABA. To address this question, we inspected 13 different Old World bipartite begomoviruses [8] for the presence of ABRE, as well as CE1 and CE3 motifs, in their presumptive BV1 promoters. The core motif of ABRE was determined to be ACGTGG or ACGTGT [23,25,36]. ABREs were also known to frequently overlap with the G-box motif with the consensus of CACGTG, which specifically interacts with two families of TFs known as basic helix-loop-helix (bHLH) and basic leucine zipper (bZIP), which are prevalent not only in plants but, also, in yeast and animals, and to which most ABA-responsive TFs belong [23,37]. As shown in Table 1, 11 of the 13 viruses had at least one copy of ABRE (shaded light blue) in their BV1 promoters, four of them had two to three ABREs. The two viruses that lacked a fully conserved ABRE, CIGMV and KuMV, nevertheless contained the related G-box motif or its highly conserved cores—CANNGT or ACGT [37] (highlighted with dotted underlines in Table 1). In summary, ABRE and the closely related G-box elements are present in the BV1 promoters of all of the 13 viruses.

In promoters of plant ABA-responsive genes, ABREs, especially when present in a single copy, frequently mediate the ABA responses in coordination with other promoter elements—among them, CE1 and CE3 [26,35]. The core motif of CE1 has been variously defined as CCACC, CCGCC, CACCG, or CGCCG in different plant genes and experimental systems [23,26,35,38,39,40]. Importantly, CE1 and, also, CE3, are functional in both strands of the promoter DNA. As shown in Table 1, at least one CE1 motif (shaded purple) was identified in eight of the 13 promoters. Indeed, if we included the GGTGG (CCACC in the complementary strand) motif that overlapped with ABRE in the first five promoters, we would end up having 11 of the 13 promoters with various CE1 core motifs. Interestingly, the GGTGG element also frequently overlapped with the conserved late element (CLE; double-underlined in Table 1) found in the promoters of many geminiviral late genes, including both AV1 and BV1 [41,42], which further implicates the CE1 motifs in the transcriptional activation of BV1 genes. 

We next analyzed the 13 BV1-promoter sequences for CE3 motifs using the GTGTC and CGCGTG core motifs (painted green in Table 1) reported by others [26,35,38,39]. Note that the GTGTC motifs that overlapped with the ACGTGT, one of the ABRE consensuses, were not counted as CE3. This analysis showed that six of the 13 promoters contained CE3 motifs. Altogether, we found that all 13 BV1 promoters analyzed contained ABA-responsive promoter elements, and 12 out of 13 had more than one element. These findings are highly consistent with the involvement of ABA-responsive TFs in the activation of BV1 genes in these viruses. 

### 3.5. Exogenous ABA Has No Effect on P_BV1_ Promoter Activity or AC2-Mediated Transcriptional Activation

**Figure 4:**
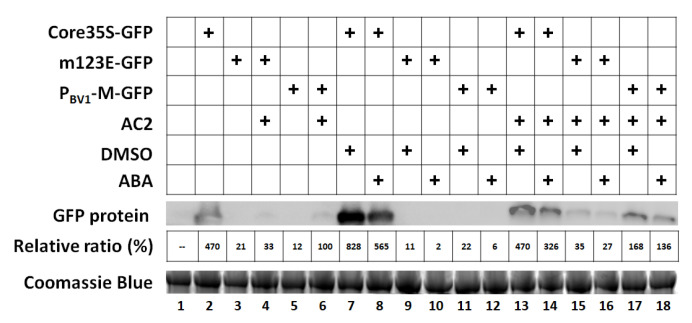
Exogenous ABA has no effect on the PBV1 promoter activity or its responsiveness to AC2 activation. Western blotting was used to detect the levels of the GFP protein in *N. benthamiana* leaves infiltrated with various combinations of Agrobacterium strains, with or without ABA (shown on top). Each protein sample was extracted from four separate leaf sections subjected to the same treatment, taken from four different plants. The relative ratios were averages of three independent sets of experiments, with the band intensity value of P_BV1_ + AC2 set at 100%. DMSO: dimethyl sulfoxide.

We earlier revealed a definitive role for ABREs in the AC2-mediated transcriptional activation of the MYMV P_BV1_ promoter. We also found that three types of ABA-responsive promoter motifs: ABREs, CE1, and CE3, were prevalent in BV1 promoters of many Old World bipartite begomoviruses. We hence wondered whether applying exogenous ABA could stimulate the transcription from a BV1 promoter, with or without AC2. For this experiment, we returned to using the MYMV P_BV1_ and its m123E mutant, in which all three ABREs were mutated. We also included an additional control in which the transcription of GFP mRNA was driven by the core (the last 99 nt) of the CaMV 35S promoter (Core35S) that did not contain an ABRE. Notably, the transcription driven by Core35S was close to five times stronger than the AC2-activated P_BV1_ and approximately 40 times stronger than P_BV1_ alone (Figure 4, lanes 2, 5, and 6), verifying P_BV1_ as an extremely weak promoter in the absence of AC2 activation. Additionally notable is that the activity of the Core35S promoter was repressed by AC2 by approximately 40%, regardless of the presence of exogenous ABA (Figure 4, compare lanes 7 and 8 with 13 and 14). Additionally, DMSO, which was used to dissolve ABA and, hence, was included as a solvent control, appeared to stimulate the Core35S transcription, but its effect seemed to be attenuated by ABA (compare lanes 7 with 8 and 13 with 14). 

However, ABA failed to elicit a significant stimulation of the transcription from the P_BV1_ promoter, regardless of the presence of AC2. Although the basal transcription experienced a slight increase for the samples in lanes 15 and 16 (compared with lane 5), this was likely due to the stimulatory effect of DMSO. Similar conclusions could be drawn for the samples in lanes 17 and 18 (compared with lane 6). In summary, although the three ABREs within P_BV1_ were clearly needed for AC2-mediated transcriptional activation, the corresponding TFs were not upregulated by the ABA treatment in *N. benthamiana* leaf cells (see Discussion). 

## 4. Discussion

The question of how AC2, a protein encoded on the DNA-A of bipartite begomoviruses, transactivates the late genes encoded on the DNA-B, a separate genome segment, is important to resolve, as the answer could offer insights on how genimiviruses coordinate the expression of early and late genes to balance the optimal genome replication with an efficient whole-plant spread. It could also inform us of novel strategies on controlling these viruses. The findings made using the TGMV model strongly suggest that AC2/AL2 acts on the BV1/BR1 promoter by recruiting PPD2, a putative TF of the plant host that confers the DNA-binding specificity [14,18,19]. However, whether the same TF could also be exploited by AC2 of other, similar viruses was not reported. In the current study, we found that the BV1 promoters of the MYMV and multiple other bipartite begomoviruses of the Old World lineage were enriched for conserved promoter elements characteristic of ABA-responsive genes of diverse plants [23,26,38]. Furthermore, eliminating the three ABREs in the MYMV BV1 promoter led to a near complete loss of the AC2 responsiveness. Together, our results strongly suggest that TFs of the ABA-responsive pathways could be candidates for AC2 recruitment. Since many TFs implicated in the ABA pathways are already known, this study substantially narrows down the search for TFs that interact with the MYMV AC2 in future investigations. 

While the importance of ABA-responsive promoter elements in BV1 expression has not been recognized before, the involvement of the closely related G-box motif in the transcription of AC1/AL1 (Rep) has been reported [43]. Separately, ABA-responsive genes were found to be induced in tomato plants infected with the tomato yellow leaf curl Sardinia virus [44]. ABA has also been found to enhance the plant defense against RNA viruses by inducing the expression of RNA-silencing pathway genes [45]. These studies are consistent with the active role of ABA signaling in plant responses to virus infections. Conversely, it is conceivable that some geminiviruses may evolve to turn the ABA-mediated defense signaling to their own advantage by coopting it to activate the expression of late genes of the viruses. 

An unexpected finding of our experiments is that the MYMV BV1 promoter is very weak. Although we expected to see low transcription without AC2 activation, we were nevertheless surprised to realize that its activity was still many folds weaker than the Core35S promoter upon AC2 activation. Several reasons might account for this low activity. First, the activity detected with our system may only account for a fraction of the BV1 transcription in actual MYMV infections. This is because, in actual infections, BV1 and other late genes are expressed after viral replication from the many progeny genomes newly synthesized in the cells. As a result, BV1 transcription is expected to occur simultaneously on many copies of DNA-B. By contrast, the amount of nonreplicating DNA templates in our nonreplicating system is expected to be very limited. Second, optimal BV1 transcription may demand an ideal host cell environment not provided with our experimental system. The MYMV is a virus of legume plants. Hence, the TFs accessible to AC2 in legumes may differ substantially from their orthologs in *N. benthamiana*, making transcriptional activation less efficient in our system. Consistent with this idea is the fact that the BV1 promoter of the SYMV exhibited much higher basal transcription than the MYMV BV1 promoter in our assay, suggesting virus- and host-dependent effects. 

The lack of response to ABA treatment by the MYMV BV1 promoter is also noteworthy, given the requirement of ABREs for its activation by AC2. This could be explained if the particular ABA-responsive TF(s) were present in *N. benthamiana* leaf cells at low levels, but their corresponding genes were not responsive to ABA in these particular cells. This scenario is possible, because ABA responses are known to be highly coordinated, often occurring in dividing cells at root and shoot growth points [24]. Consistent with this, geminiviruses are known to be mostly phloem-limited, primarily infecting cells of vascular bundles where they induce active division of the infected cells. By contrast, the *N. benthamiana* cells we used were mostly nondividing. Nevertheless, our system did detect a substantial level of AC2 activation, permitting the interrogation of the ABREs in the MYMV BV1 promoter. In conclusion, our study revealed a previously unrecognized class of promoter elements that mediate the AC2 transcriptional activation of a BV1 promoter of a bipartite begomovirus and paved the way for the identification of the specific TF(s) involved in the process.

## Figures and Tables

**Table 1:** ABA-responsive ABRE, CE1, and CE3 motifs within BV1 promoters of bipartite begomoviruses.

Virus_DNA-B Accession	DNA-B nt #26-175 (150 nt), Conserved Promoter Elements Denoted
MYMV-Vigna [KA22] _AJ132574.1	TGGGTATTAGAGCACGTGGGTGGTCCCTCTATTACGTGGCGCGACGTGGATTCTCGCTCGGAGCTTGTTTATTGAACGACAACTTCGACTAACAGGTAACCGGTTTGGTTACCTTTCGTACATGGACAAATTTGTCTTTTCCTCAAAAAG
MYMV-Vigna [KA34] _AJ439057.1	TGGGTATTAGAGCACGTGGGTGGTCCCTCTATTACGTGGCGCGACGTGGATTCTCGCTCGGAGGTTGTTTATTGAACGACAACTTGGACTAACAGGTAACCGGTTTGGTTACCTTTCGTACATGGACAAATTTGTCTTTTCCTCAAAAAG
SYMV_AJ582267.1	TGGGTATTTCGCCCACGTGGGTGGTCCCTCTAAGACGTGGCGCACTCTGGAACCGCGCTGGGAGGTTATTTAAGACTTTGTCTACTTGTGGTAACAGGTAACCGGCTAAGTGACCGGTTGGAAAGCGTGCCTTTCCCACCCCTGGCAAAT
MYMIV_AY271894.1	TCGGTTTTAGAGCACGTGGGTGGTCCCTCTAGTACGTGGCGCGCTCTGGAGTCTCGCTCGAAGCTTGTTTATCGAACGACTACTTAGAGTTACAGGTAACCGGATAGGTGACCGATCGTACATGGACAAATTTGTCTTTTCCTCAAAAAG
HYMV_AJ627905.1	GACCTGGATCACTCACGTGGGTGGTCCCGCTCATCGTGGCGCAACGAGGAGTCTCGTTGGGAGTTTTTATAATGGGTTACTACTTGGGAGAAGTGGGGAAACCGGAAGCTACCGCCGCAAATCGAGAAATTTGCTTTTAATGCGAAATTA
SLCMV_AJ579308.1	GTGGTGGCCCCCCCCACGTGGGGATGTCCCCCTCTCACAACGCTCACTAGAAGGTTCAACATGTTGGTGGCCCCACGATGTTGTTTATAACGTCTATAACGTTTGAGACTCGAAGCTTGTGATGCCACGTATGCGTTATTAGTACTTCGT
ICMV_AJ314740.1	GTGGTGGACCCCTCCCCCACGTGGAGATGTCCCCCACTCAGAACGCTCACTAGGAGGTTCAACATGTTGGTGGCCCCACGATGTTGTTTATAACGTCTATAACGTCTGAGACTCCAAGCTTGTGATGCCACGTATGTGTTATTTGTACTT
ClGMV_DQ641693.1	TATAGTATGTGTCATGTGTGGGTCCCATGTCACGTGATTTTGTACCTTTCTGTCCCCCAGCTAGCCGACAAAGTGGGACCCACTCACGTGATTAGCACTTGCCTTGTACCTTCCTTCTGTGTACGTTCTGGTCAAAAAGACTAGTATACC
KuMV_DQ641691.1	TGCGGTGTGGTCCCCCCGCCCA**TG**TGCTTTCAATCTCGTCCGCACGTTTTTTTTAGTCTTCGCGCGTGGTGTGAATGCGCCGTAATGCGCTTGCCGCAAATAACGTTATAACTGCTTTAATTTGAATTTCTTTAAATTATGTGGAATACC
SLCCV_AM260207.1	CTCTAAATCTGGCCGTCTATTTTCTATAAATGCGCATCAAACGAGTGCGATGAACAACACGTGTCCTATGTGTCATCCTTTACTAAAGGAAAGGATTGTACGATATCGAAATTTGCACATGGTGGACCCCAGTGACTAGATATGCAATAT
SLCPHV_AB085794.1	CTTCAAATCTGTCCGTCTATTTTGTACAAAAGCTTATCAAGGTGCTGCGATGCACAACACGTGTCCTCTATTACATCCTTTACTAAAGTAAAGGTTTGTACGATTTCCAAGTGCCGCACATGGTGGTCCCCAGTGACTAGATATGCAATA
LYMV_AF509740.1	TCTCCCCACAGTCGTTGATTTGTTCTAAAGGCGCGCACGATTGCATGTTTTCGCAACACGTGTCCTCTTATGATTACTTTATGAAAATAAAGTTAGCTGTGTTCTGAAATTTCGCACATGGTGGTCCCCCAGATACTAGATAGAAATATC
ToLCNDV_AJ875158.1	CCCTATCCTGACCGTTGCTGTGTAATCATTGCACCAAGTTAGTCATCCGATTTGCAACACGTGTATCCCACTAACAGACTTTATGCAAATAAATGTCCGATATCTGTGTCGACAATGCTTGTGTGTTCCCCTTATATCTTGTCGTACCCC
ToLCuGuV_AY190291.1	CCTTATCTTGACCGTTGCTGCGTAATCATTGCACCAAGTTACTCATCCGATTTGCAACACGTGTATCCCACTAGCAGACTTTATGCAATTAAATGTCTGATATCTGTGTGTACAATGCATATGTGTTCCCCTTATATCTTGTCGTACCCC

**Notes:** 1. The highly conserved core motifs of ABRE, CE1, and CE3 are: ABRE: ACGTGG, ACGTGT; CE1: CCACC, CCGCC, CACCG, CGCCG; CE3: GTGTC, CGCGTG. The well-documented G-box motif (CACGTG), as well as two variations of core motifs of G-box (CANNTG and ACGT), are highlighted with dashed underlines. Finally, the conserved late element (CLE, GTGGTCCC) found in the promoters of late genes in many begomoviruses are highlighted with double underline. 2. Virus acronyms: MYMIV—mungbean yellow mosaic India virus, HYMV—horsegram yellow mosaic virus, SLCMV—Sri Lankan cassava mosaic virus, ICMV—Indian cassava mosaic virus, ClGMV—Clerodendrum golden mosaic virus, KuMV—Kudzu mosaic virus, SLCCV—squash leaf curl China virus, SLCPHV—squash leaf curl Philippines virus, LYMV—loofa yellow mosaic virus, ToLCNDV—tomato leaf curl New Delhi virus, ToLCuGuV—tomato leaf curl Gujarat virus.
